# Supplementary material for: Synthesis of Network Polymers by Means of Addition Reactions of Multifunctional-Amine and Poly(ethylene glycol) Diglycidyl Ether or Diacrylate Compounds
Source: Polymers (Basel). 2020 Sep 8;12(9):2047. doi: 10.3390/polym12092047 (PMC7570363; doi:10.3390/polym12092047)
Supplement: Supplementary file 1 [file polymers-12-02047-s001.pdf]

Supplementary Material

Synthesis of network polymers by means of addition reactions of multifunctional-amine and poly(ethylene glycol) diglycidyl ether or diacrylate compounds

Naofumi Naga [1,2*], Mitsusuke Satoh [2], Kensuke Mori [1], Hassan Nageh [3], and Tamaki Nakano [3,4]

[1] Department of Applied Chemistry, College of Engineering, Shibaura Institute of Technology, 3-7-5 Toyosu, Koto-ku, Tokyo 135-8548, Japan

[2] Graduate School of Science & Engineering, Shibaura Institute of Technology, 3-7-5 Toyosu, Koto-ku, Tokyo 135-8548, Japan

[3] Institute for Catalysis and Graduate School of Chemical Sciences and Engineering, Hokkaido University, N 21, W 10, Kita-ku Sapporo 001-0021, Japan

[4] Integrated Research Consortium on Chemical Sciences, Institute for Catalysis, Hokkaido University, N 21, W 10, Kita-ku Sapporo 001-0021, Japan

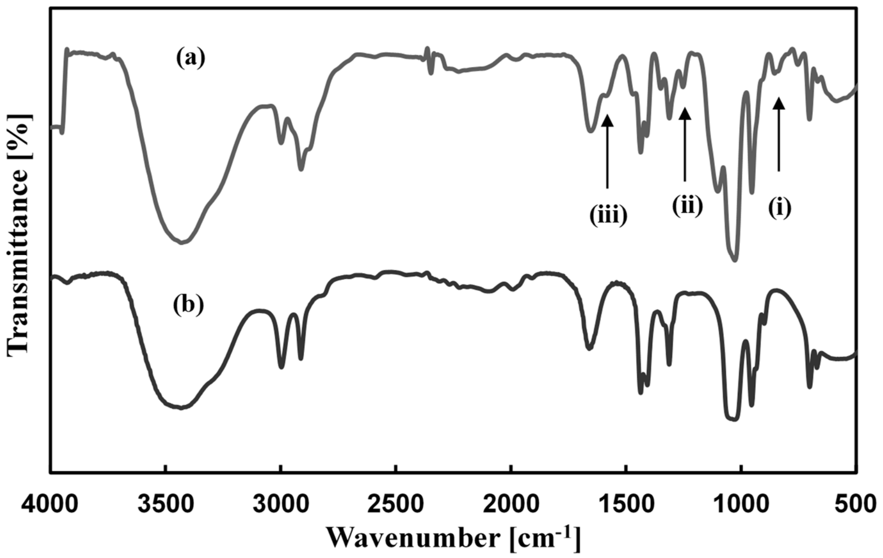

Figure S1. FT-IR spectra of PEI-PEGDE400 reaction system Case 2, (a) before reaction, and (b) after reaction at 90 °C for 24 h, solvent: DMSO, monomer concentration: 30 wt%.

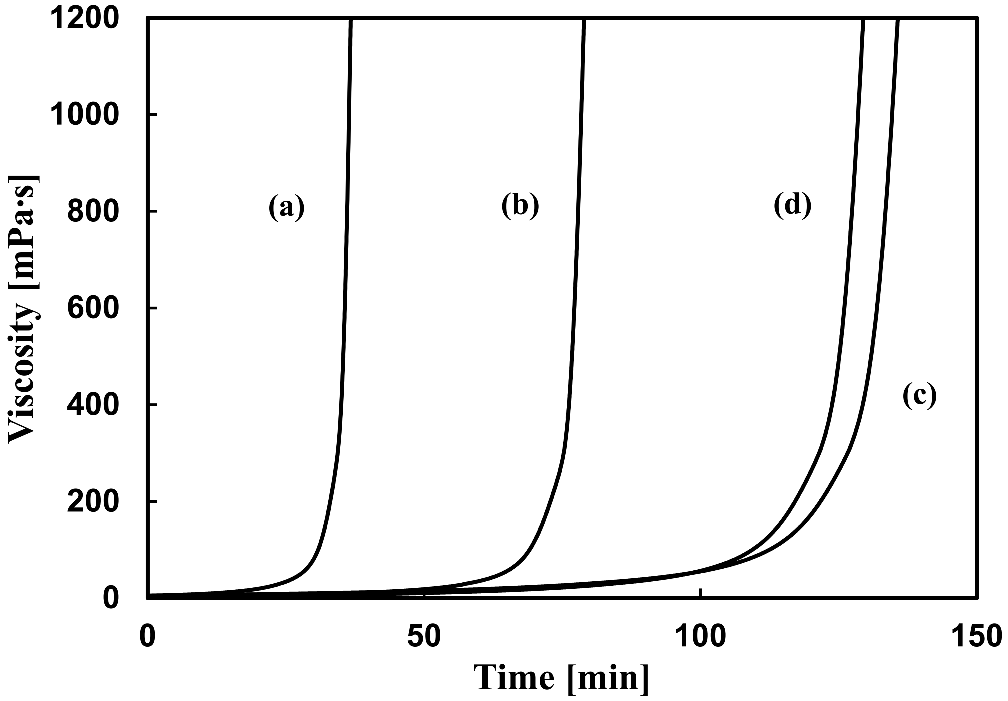

Figure S2. Time evolution of viscosity of PEI-PEGDE400 reaction systems: (a) Case 1, (b) Case 2, PEI-PEGDE1000 reaction system: (c) Case 1, DETA-PEGDE400 reaction system: (d) Case 1, solvent: H_2O, monomer concentration: 30 wt%.

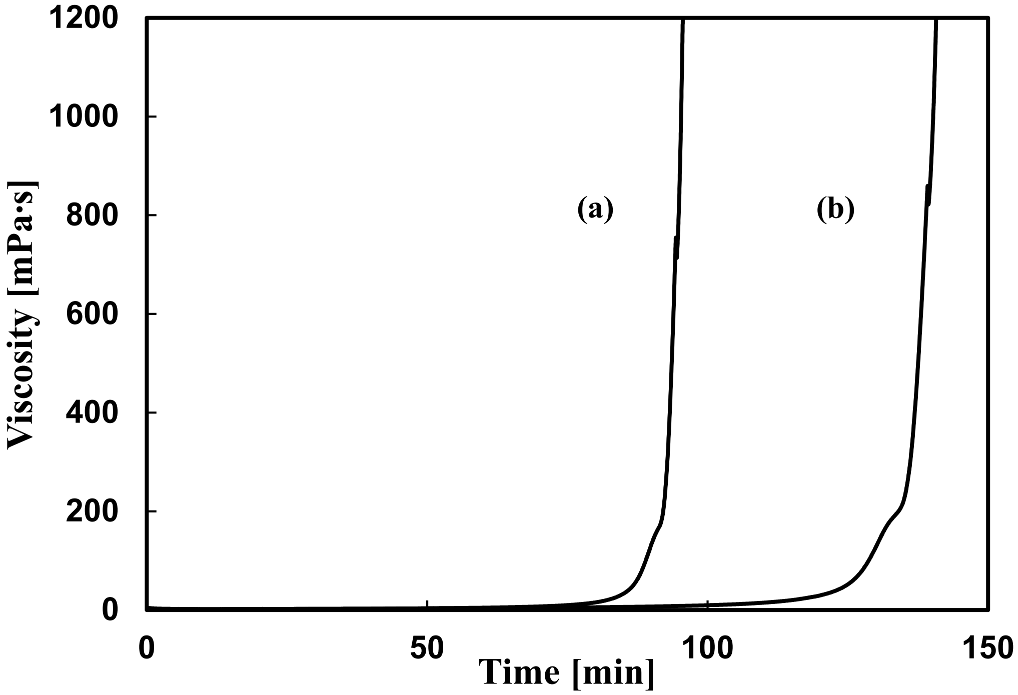

Figure S3. Time evolution of viscosity of PEI-PEGDE400 reaction systems: (a) Case 1, (b) Case 2, solvent: DMSO, monomer concentration: 30 wt%.

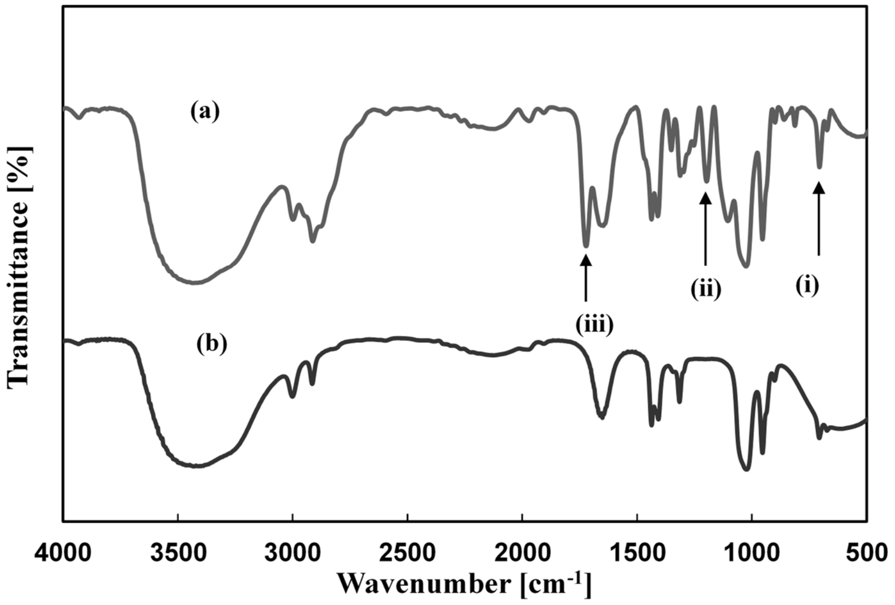

Figure S4. FT-IR spectra of PEI-PEGDA400 reaction system (a) before reaction, and (b) after reaction, Case 2, solvent: DMSO, monomer concentration: 30 wt%.

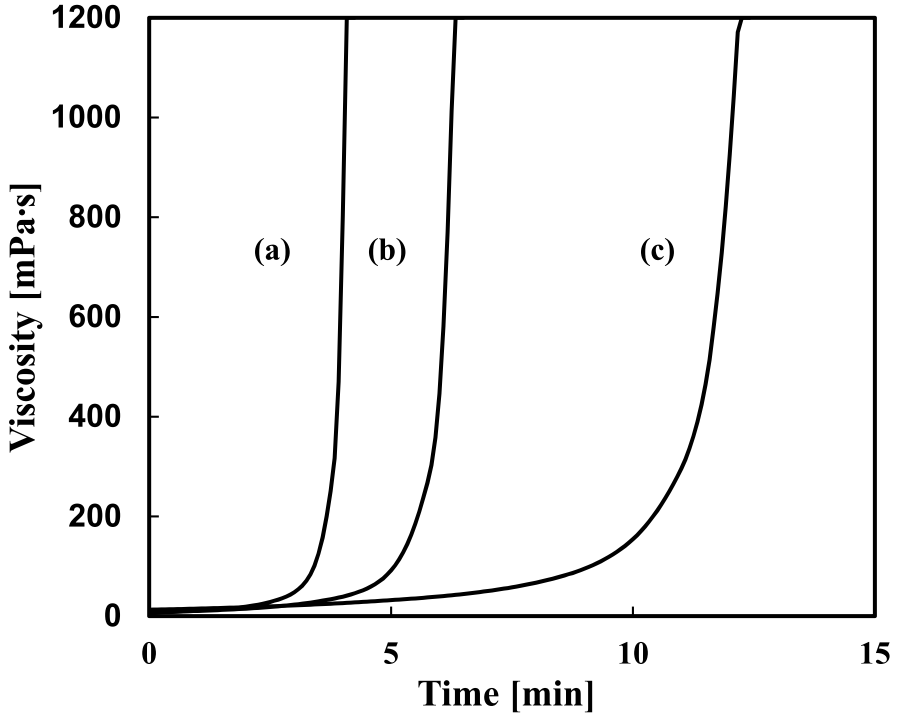

Figure S5. Time evolution of viscosity of (a) PEI-PEGDA200 reaction system Case 1, (b) PEI-PEGDA400 reaction system Case1, (c) PEI-PEGDA600 reaction system Case 1, solvent: DMSO, monomer concentration: 30 wt%.

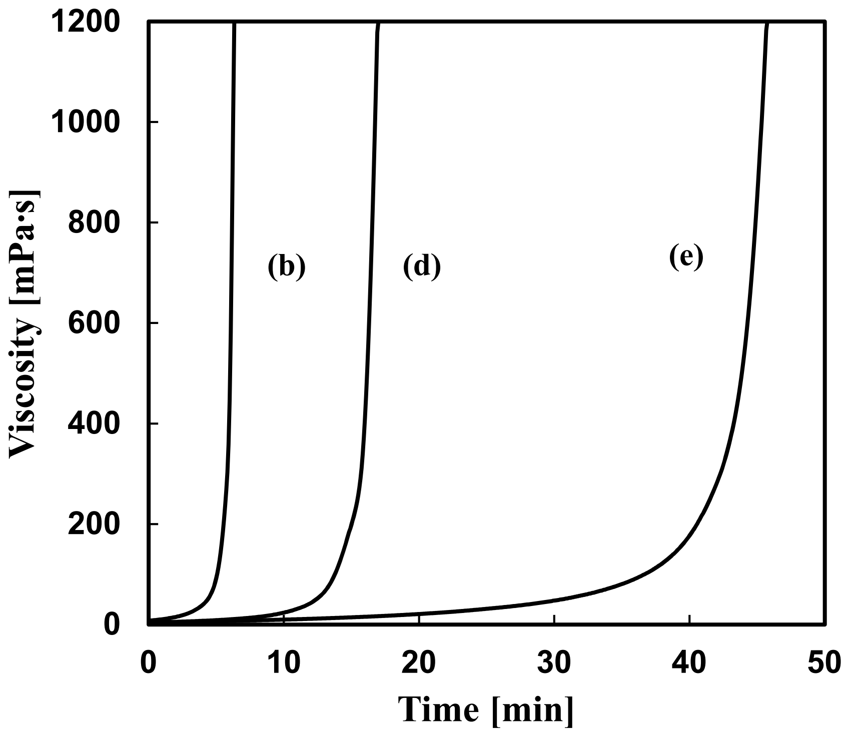

Figure S6. Time evolution of viscosity of PEI-PEGDA400 reaction system: (b) Case 1, (d) Case 2, DETA-PEGDA400 reaction system: (e) Case 1, solvent: DMSO, monomer concentration: 30 wt%.

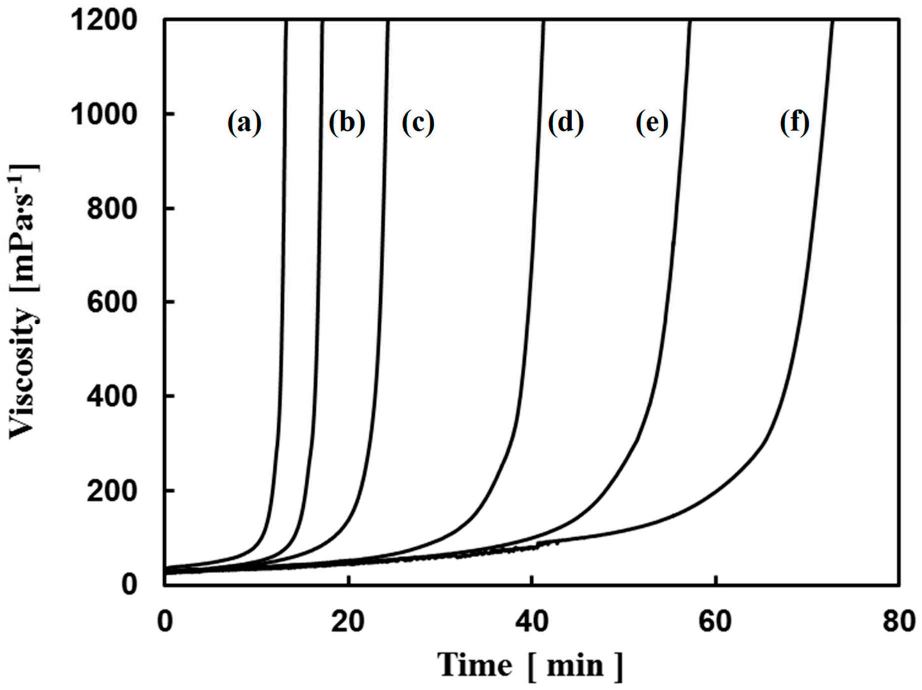

Figure S7. Time-evolution of (a) PEI-PEGDA400, (b) PEI-PEGDA600 and (c) PEI-PEGDA1000 reaction systems Case 1, (d) PEI-PEGDA400, (e) PEI-PEGDA600 and (f) PEI-PEGDA1000 reaction systems Case 2, solvent: EtOH, monomer concentration: 30 wt%.

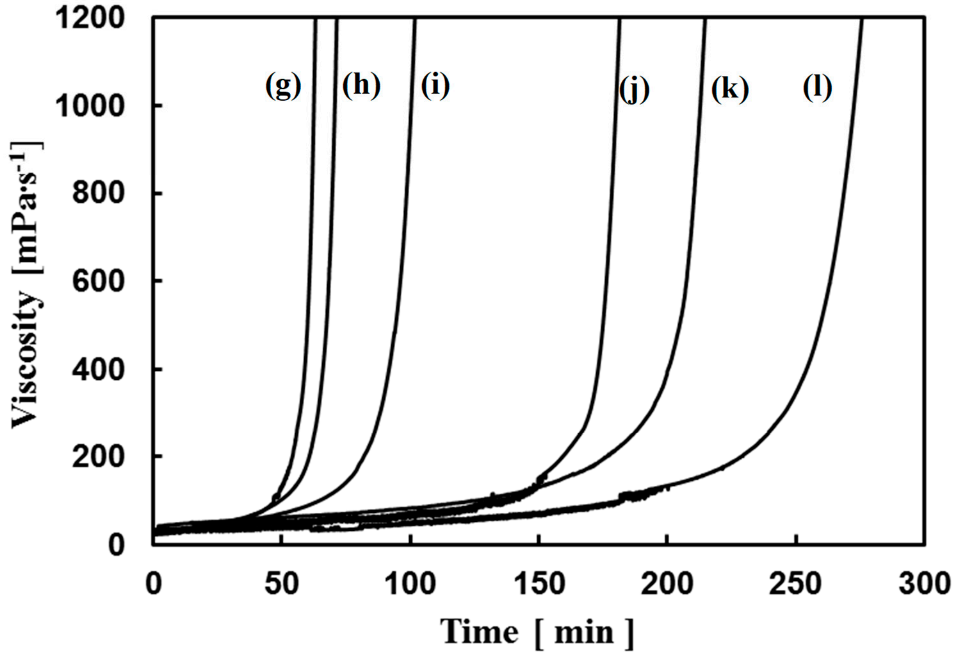

Figure S8. Time-evolution of viscosity of DETA-PEGDA reaction systems, (g) DETA-PEGDA400, (h) DETA-PEGDA600, and (i) DETA-PEGDA1000, Case 1, (j) DETA-PEGDA400, (k) DETA-PEGDA600, and (l) DETA-PEGDA1000, Case 2, solvent: EtOH, monomer concentration: 30 wt%.

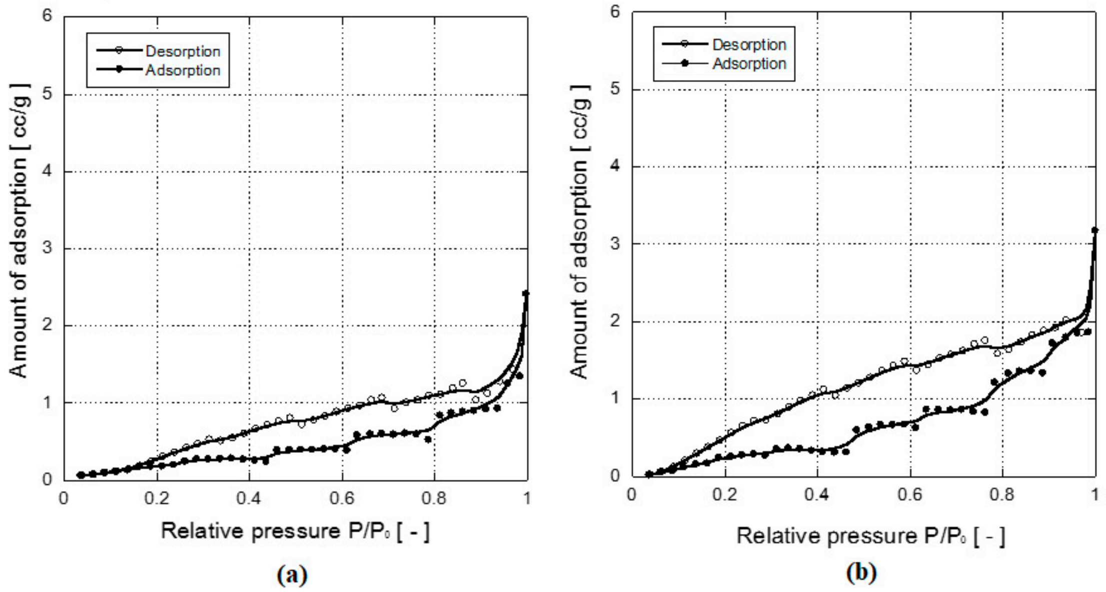

Figure S9. Adsorption isotherm of (a) PEI-PEGDA200 and (b) DETA-PEGDA200 porous polymers, monomer concentration in the reaction solution: 30 wt%, feed molar ratio: [PEI]/[PEGDA200] = 2/14 mol/mol, [DETA]/[PEGDA200] = 2/5 mol/mol (Case 2).

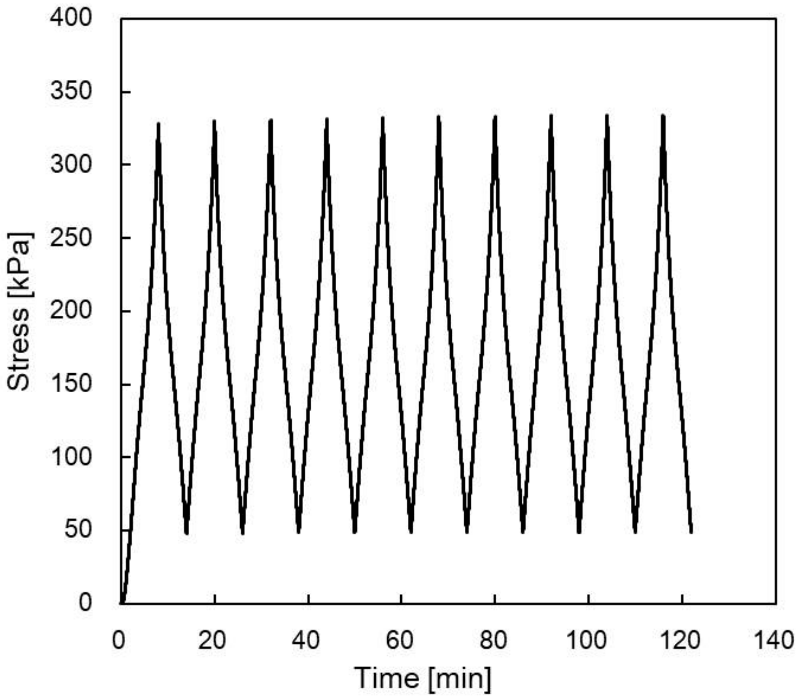

Figure S10. Cycle test of DETA-PEGDA200 porous polymer, monomer concentration in the reaction solution: 30 wt%, feed molar ratio of DETA/PEGDA200: 2/5 Case 2), sample size: 10 mm cube, upper point: 4 mm – lower point: 1 mm, compression and release rate: 0.5 mm/min, cycle: 10.

Table S1. Affinity of PEG and solvents calculated by Hansen solubility parameters.

Solvent	dD_2 $MPa^{1/2}$	dP_2 $MPa^{1/2}$	dH_2 $MPa^{1/2}$	R_a [a] $MPa^{1/2}$
hexane	14.9	0	0	15.3
EtOH	15.8	8.8	19.4	19.2
acetone	15.5	10.4	7.0	10.2
toluene	18.0	1.4	2.0	10.6
water	15.5	16.0	42.3	41.3
DMSO	18.4	16.4	10.2	9.9
dichloromethane	17.0	7.3	7.1	8.6
chloroform	17.8	3.1	5.7	9.8

[a] Hansen distance: $R_a = \{4*(dD_1 - dD_2)^2 + (dP_1 - dP_2)^2 + (dH_1 – dH_2)^2\}^{1/2}$

PEG: $dD_1 = 20.0$, $dP_1 = 11.2$, $dH_1 = 2.3$ ($MPa^{1/2}$)
